# Histogram analysis based on multi-parameter MR imaging as a biomarker to predict lymph node metastasis in T3 stage rectal cancer

**DOI:** 10.1186/s12880-021-00706-0

**Published:** 2021-11-22

**Authors:** Yang Zhou, Rui Yang, Yuan Wang, Meng Zhou, Xueyan Zhou, JiQing Xing, Xinxin Wang, Chunhui Zhang

**Affiliations:** 1 Department of Radiology, Harbin Medical University Cancer Hospital, No. 150, Haping Road, Nangang District, Harbin, 150001 Heilongjiang Province China; 2 Department of Gastrointestinal Medical Oncology, Harbin Medical University Cancer Hospital, No.150 Haping Road, Nangang District, Harbin, 150081 Heilongjiang Province China; 3 School of Technology, Harbin University, Harbin, Heilongjiang Province China; 4 Department of Physical Education, Harbin Engineering University, Harbin, 150001 Heilongjiang Province China

**Keywords:** Rectal cancer, LNM, Multi-parametric magnetic resonance imaging, Histogram, Nomogram

## Abstract

**Background:**

Preoperative identification of rectal cancer lymph node status is crucial for patient prognosis and treatment decisions. Rectal magnetic resonance imaging (MRI) plays an essential role in the preoperative staging of rectal cancer, but its ability to predict lymph node metastasis (LNM) is insufficient. This study explored the value of histogram features of primary lesions on multi-parametric MRI for predicting LNM of stage T3 rectal carcinoma.

**Methods:**

We retrospectively analyzed 175 patients with stage T3 rectal cancer who underwent preoperative MRI, including diffusion-weighted imaging (DWI) before surgery. 62 patients were included in the LNM group, and 113 patients were included in the non-LNM group. Texture features were calculated from histograms derived from T2 weighted imaging (T2WI), DWI, ADC, and T2 maps. Stepwise logistic regression analysis was used to screen independent predictors of LNM from clinical features, imaging features, and histogram features. Predictive performance was evaluated by receiver operating characteristic (ROC) curve analysis. Finally, a nomogram was established for predicting the risk of LNM.

**Results:**

The clinical, imaging and histogram features were analyzed by stepwise logistic regression. Preoperative carbohydrate antigen 199 level (*p* = 0.009), MRN stage (*p* < 0.001), _T2WI_Kurtosis (*p* = 0.010), _DWI_Mode (*p* = 0.038), _DWI_CV (*p* = 0.038), and _T2-map_P5 (*p* = 0.007) were independent predictors of LNM. These factors were combined to form the best predictive model. The model reached an area under the ROC curve (AUC) of 0.860, with a sensitivity of 72.8% and a specificity of 85.5%.

**Conclusion:**

The histogram features on multi-parametric MRI of the primary tumor in rectal cancer were related to LN status, which is helpful for improving the ability to predict LNM of stage T3 rectal cancer.

**Supplementary Information:**

The online version contains supplementary material available at 10.1186/s12880-021-00706-0.

## Background

The treatment of locally advanced rectal cancer includes neoadjuvant chemoradiotherapy (nCRT) and total mesorectal excision (TME). LNM is an important prognostic factor for local recurrence and distant metastasis [1]. The European Society for Medical Oncology (ESMO) guidelines recommend that low-risk T3N0 patients do not need preoperative nCRT and only need TME. For most patients with T3N1-2 cancer, preoperative nCRT is necessary, achieving tumor degradation and reducing the risk of postoperative recurrence [2]. Therefore, the preoperative identification of lymph node status in patients with T3 rectal cancer plays an important role in guiding treatment decision-making. At present, high-resolution MRI is the standard method for evaluating LNM of rectal cancer. However, the accuracy of using MRI morphological criteria such as diameter, shape, boundary, and signal heterogeneity to assess the status of lymph nodes is unsatisfactory [3–5].

Texture analysis can reflect tumor heterogeneity and assist doctors in diagnosis by extracting many quantitative features from the region of interest of medical images [6]. Features extracted from medical images can be defined as first-order, second-order, or higher-order features [7]. Texture analysis based on first-order statistics of tumor images is called histogram analysis, which only reflects the distribution of voxel gray intensity and has no spatial information or relationship between voxels [8, 9]. Histogram analysis based on medical images has been proved to be helpful for early non-invasive identification of tumor heterogeneity, prediction of LNM, evaluation of curative effect, and prognosis of the patients [10–13].

However, the few articles about lymph node prediction in rectal cancer mainly focus on single sequence image analysis but do not give full play to the characteristics of multi-parametric MRI, and the diagnostic ability needs to be improved. Therefore, this study takes stage T3 rectal cancer as an example to explore the predictive ability of multi-parametric MRI features in lymph node diagnosis to better guide the choice of treatment for patients with stage T3 rectal cancer.

## Methods

### Patients

**Fig. 1:**
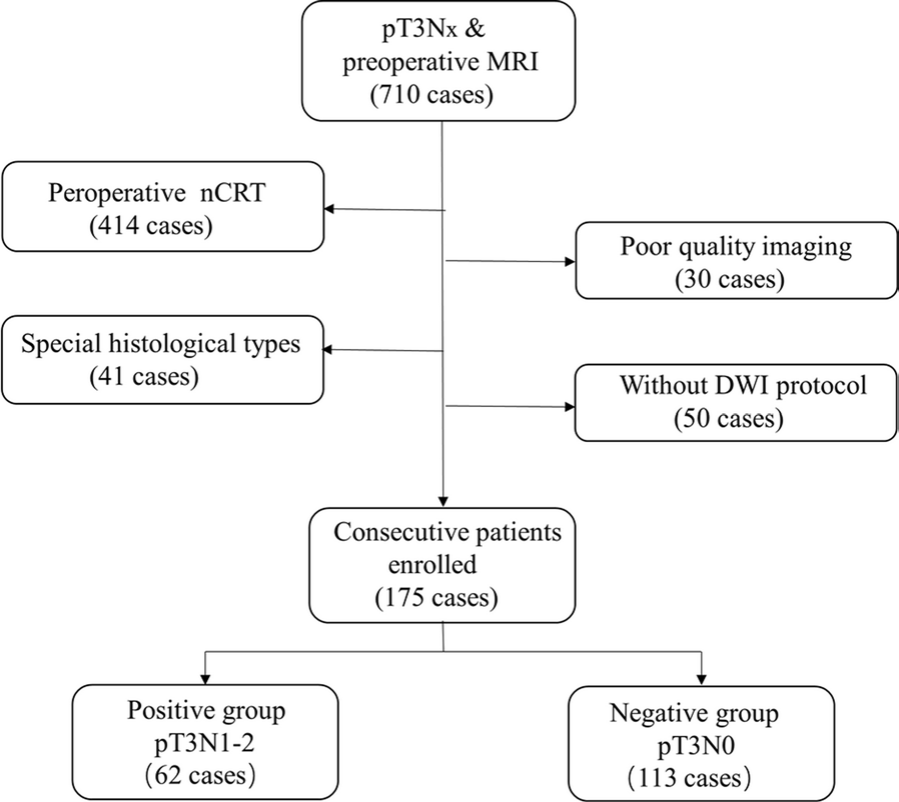
Flow chart of inclusion and exclusion criteria of the study sample

This retrospective study was approved by the Ethics Review Committee of Harbin Medical University Cancer Hospital and the need for signed informed consent was waived. We included 710 patients with rectal cancer who underwent total mesorectal resection from January 2015 to May 2017 and obtained preoperative MRI images and postoperative pathological reports. Case inclusion criteria were as follows: (1) Patients with rectal cancer confirmed by histopathology as stage T3Nx; and (2) Patients who underwent multi-parametric MRI within two weeks before the operation. Case exclusion criteria were as follows: (1) Patients with particular histological types confirmed by a histopathologist, such as mucinous adenocarcinoma or neuroendocrine tumor (n = 41); (2) Patients who received treatment such as nCRT before MRI or surgery (n = 414); (3) Patients who underwent MRI without DWI (n = 50); and (4) Patients with poor image quality (n = 30) (Fig. 1). Finally, we conducted a retrospective study of 175 patients. Clinical data, including patients’ age, height, weight, history of smoking and alcohol consumption were obtained by reviewing the medical records. Tumor marker information was obtained from laboratory-based tests in each patient. We assessed the expression status of carcinoma embryonic antigen (CEA), carbohydrate antigen (CA) 199, CA724, and α-fetoprotein (AFP), according to practical guidelines for the use of tumor markers [14].

### High resolution rectal MRI parameters

All patients underwent rectal MRI with a 3.0 T MR scanner (Achieva; Philips, Eindhoven, Netherlands), with a 16-channel torso array coil. The MRI scan sequence included T2-weighted imaging (T2WI) sequence (sagittal, short axial, and coronal position), and diffusion weighted imaging sequence (DWI) (b = 0/1000). The MRI scanning parameters were: TR/TE = 3000 ms/100 ms; number of signal frequency (NSA) = 2; layer thickness = 3 mm; layer spacing = 0.4 mm; field of view (FOV) = 240 × 240 mm. Sagittal T2WI images determined the location of the lesion, and the short axis T2WI of the lesion was located perpendicular to the long axis of the intestinal canal (TR = 3000–4000 ms; TE = 110 ms; NSA = 3; layer thickness = 3.5 mm; layer spacing = 0.2 mm). According to the location of the sagittal lesion, the coronal T2WI was located on the long axis of the parallel lesion (TR = 3824 ms; TE  = 110 ms; NSA = 3; layer thickness = 3.0 mm; layer spacing = 0.2 mm).

### Analysis of radiological features

MR images of all patients were collected by a RIS/PACS (GE Healthcare centrality, USA) system. Two abdominal radiologists (R1 and R2, with 5 and 8 years of experience in MRI diagnosis of rectal cancer) determined the stage of the patient's rectal cancer. The subjective evaluation information of patients with rectal lesions was recorded on T2WI images, including the following: (1) Lesion thickness and length: measured at the largest T2WI level. (2) Lesion location: categorized as low (0–5 cm from the anal verge), middle (5.1–10 cm from the anal verge), and high (10.1–15 cm from the anal verge); (3) mrT3 stage: classified into four categories depending on the distance between the outermost edge of the muscularis propria and the maximum extramural spread of the tumor (T3a, < 1 mm; T3b, 1–5 mm; T3c, 5–15 mm; and T4d, > 15 mm) [15]; (4) mrN stage: doctors observed and classified the size and morphological characteristics of visible lymph nodes on T2WI; the criteria for a malignant node: (1) Short axis diameter ≥ 9 mm; (2) Short axis diameter 5–8 mm and ≥ 2 morphologically suspicious characteristics; (3) Short axis diameter < 5 mm and 3 morphologically suspicious characteristics. Morphologically suspicious criteria were: round shape, irregular border, and heterogeneous signal [4]. The number of suspected lymph nodes in the region was evaluated according to the above criteria; no suspicious lymph nodes was mrN0; 1–3 suspected lymph nodes was mrN1; 4 or more suspected lymph nodes was mrN2 [16]. (5) Extramural vascular invasion (EMVI): EMVI is an extension of the tumor to the vessels in the mesorectum, resulting in wall irregularity, focal enlargement, and signal intensity (SI) of the tumor within the vessel [15]. (6) Mesorectal fascia (MRF): the shortest distance between the primary tumor of rectal cancer, suspected lymph nodes or tumor deposit, and the MRF was measured. If the measured value was < 1 mm, it was evaluated as MRF positive [15]. If there was a difference in the radiologist's opinion in the above characteristic assessment, a solution was determined through consultation.

### Histogram features extraction

All images were stored in DICOM format and input into Matlab software. Another radiologist (R3, with 3 years of experience in MRI diagnosis of rectal cancer) used Matlab software (MathWorks, Natick, MA, USA) to identify the tumor and select the region of interest (ROI), and a senior doctor (R1) supervised this. The radiologist (R3) only knew that the focus was rectal cancer and ignored the clinical and pathological features. The T2WI-ROI and DWI-ROI were obtained by delineating ROI directly along the margin of the whole tumor on the maximum cross-sections of T2W images and DW images (high b value) on the axial plane, excluding the intraluminal gas, and the obvious necrotic and cystic areas. On the basis of T2WI-ROIs we obtained T2WI texture features. Then we mapped the DWI-ROIs to T2WI and the ADC map, respectively, obtained the T2-map-ROI and ADC-ROI, and extracted texture features from these ROIs. Finally, four groups of histogram features of the T2WI-ROI, DWI-ROI, T2-map-ROI and ADC-ROI were obtained from each patient.

**Fig. 2:**
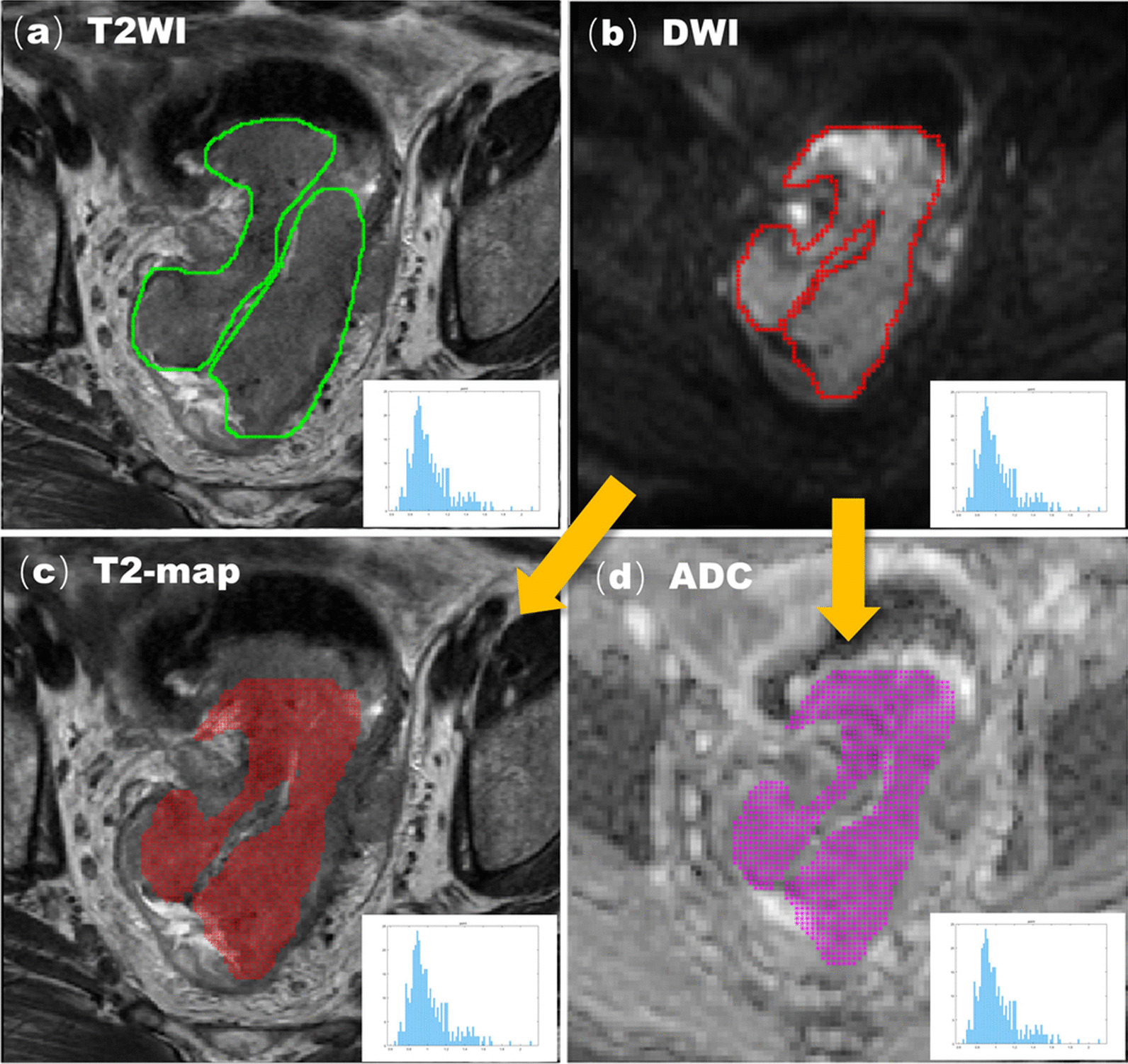
An example of manual segmentation of MRI in the primary tumor of rectal cancer. **a** T2W maximum cross-sectional view, manually sketched T2WI-ROI (green intra-line region) and its histogram of primary tumors in patients with stage T3 rectal cancer. **b** The same slice DWI image from the same patient, the manually sketched DWI-ROI (red intra-line area), and its histogram. **c** The T2-map-ROI (red mosaic region) and its histogram of T2-map were obtained by mapping DWI ROI to T2WI. **d** The ADC-ROI (magenta mosaic region) and its histogram of the ADC diagram were obtained by mapping DWI ROI to the ADC graph

The histogram of signal strength distribution was then generated in the ROI, including mean, median, maximum, minimum, standard deviation, coefficient of variation (CV), kurtosis, skewness, and mode (Fig. 2).

### Histopathological evaluation

Histopathological assessment is the gold standard for LN malignancy. The pathological report of surgically resected specimens contained a standard data set based on the 8th edition of the American Joint Commission on Cancer (AJCC) TNM staging system. Tumor differentiation, depth of invasion, and pathological N stage (pN) were obtained retrospectively. According to the pathological data, the patients were divided into the positive and negative lymph node groups.

### Statistical analysis

Medcalc (version 11.4, Medcalc, Belgium), SPSS (version 24.0 IBM, NY, USA), and R software (version 4.0.3, The R Project) were used for statistical analysis. Medcalc was used to draw the receiver operating characteristic (ROC) curve of all histogram features, and the value corresponding to the maximum Youden index was the best diagnostic cutoff value. These values were used to transform histogram features into classification variables. Statistical analysis of the clinical, doctor's diagnosis and histogram characteristics was carried out using SPSS software. The continuous variables were compared by the independent sample t-test or Mann Whitney U test, and the classified variables were compared by the chi-square test. The statistically significant characteristics were analyzed by univariate and multivariate logistic regression analysis to determine the independent predictors of LNM. ROC curve analysis was used to determine the sensitivity, specificity, positive predictive value (PPV), negative predictive value (NPV), and area under the curve (AUC) of the combined model and the independent predictive factor model. *p* < 0.05 was considered statistically significant. The nomogram was drawn by R software. In addition, the calibration plots were obtained by internal verification of the model by the bootstrap method. Finally, the clinical decision curve was drawn.

## Results

### Patient characteristics

One hundred and seventy-five patients with stage T3 rectal cancer confirmed by postoperative pathology were enrolled in the study. The clinical and pathological data on lymph nodes in the non-LNM group (n = 113) and LNM group (n = 62) are shown in Table 1. Except for the level of CA199 (*p* = 0.007), there was no significant difference in clinical characteristics between the two groups.

**Table 1:** Comparison of clinical data between the LNM and non-LNM groups

Clinical parameters	LNM	Non-LNM	*p* value
Sex			
Male	37 (59.7%)	76 (67.3%)	0.316
Female	25 (40.3%)	37 (32.7%)	
Age (year)	58.86 ± 10.52	62.09 ± 10.72	0.056
Height (cm)	165.50 (160.0–174.25)	168.0 (162.0–172.0)	0.880
Weight (kg)	66.50‍ (59.75–72.0)	65.0‍ (59.0–73.0)	0.851
Smoking			
No	38 ‍(61.3%)	63 ‍(55.8%)	0.478
Yes	24 ‍(38.7%)	50 ‍(44.2%)	
Alcohol			
No	40‍ (64.5%)	74 ‍(65.5%)	0.897
Yes	22 ‍(35.5%)	39‍ (34.5%)	
CEA			
< 5 ng/mL	31 ‍(51.7%)	73‍ (65.2%)	0.084
≥ 5 ng/mL	29‍ (48.3%)	39 ‍(34.8%)	
CA199			
< 37 U/mL	50 ‍(84.7%)	106 ‍(96.4%)	**0.007**
≥ 37 U/mL	9‍ (15.3%)	4‍ (3.6%)	
CA724			
< 6 U/mL	42 ‍(80.8%)	73‍ (85.9%)	0.429
≥ 6 U/mL	10 ‍(19.2%)	12 ‍(14.1%)	
AFP			
< 25 ng/mL	30 ‍(100.0%)	61‍ (96.8%)	0.324
≥ 25 ng/mL	0	2‍ (3.2%)	
Histologic grades			
Poor	1 (1.7%)	2 (1.8%)	0.914
Moderate	58 (96.6%)	107 (95.5%)	
Well	1 (1.7%)	3 (2.7%)	

Continuous variables are presented as mean ± standard deviation. Categorical variables are presented as n (%)

LNM, lymph node metastasis; CEA, carcinoembryonic antigen; CA199, carbohydrate antigen 199; CA724, carbohydrate antigen 724; AFP, alpha-fetoprotein; *p* < 0.05 are shown in bold

### Radiological findings

The imaging features in the lymph node positive and lymph node negative groups are shown in Table 2. There were significant differences in mrN stage (*p* < 0.001) and MRF (*p* = 0.008) between the two groups, but there was no significant difference in tumor thickness (*p* = 0.255), length (*p* = 0.306), tumor location (*p* = 0.325), extent of invasion (*p* = 0.997), mrT stage (*p* = 0.212) and EMVI (*p* = 0.089) between the two groups.

**Table 2:** Comparison of radiological characteristics between the LNM and non-LNM groups

Imaging features	LNM	Non-LNM	*p* value
Tumor location			
Lower	25 ‍(40.3%)	46‍ (40.7%)	0.325
Middle	27 ‍(43.5%)	39 ‍(34.5%)	
Upper	10‍ (16.1%)	28 ‍(24.8%)	
Length (mm)	49.00 ‍(41.0–59.0)	45.0‍ (40.0–54.0)	0.306
Thickness (mm)	13.00 (11.0–16.0)	13.0 (10.0–16.0)	0.255
Invasion extent			
1/4–1/2	7 ‍(11.3%)	13 ‍(11.5%)	0.997
1/2–3/4	30‍ (48.4%)	54 ‍(47.8%)	
> 3/4	25 ‍(40.3%)	46 ‍(40.7%)	
mrT stage			
3a	1 ‍(1.6%)	3 ‍(2.7%)	0.212
3b	36‍ (58.1%)	71 ‍(62.8%)	
3c	17 ‍(27.4%)	17 ‍(15.0%)	
3d	8 ‍(12.9%)	22‍ (19.5%)	
mrN stage			
N0	26 ‍(41.9%)	98 ‍(86.7%)	** < 0.001**
N1	26‍ (41.9%)	15 (13.3%)	
N2	10‍ (16.2%)	0	
mrEMVI			
Negative	55‍ (88.7%)	107 ‍(95.5%)	0.089
Positive	7‍ (11.3%)	5 ‍(4.5%)	
MRF			
Negative	45 ‍(72.6%)	100 ‍(88.5%)	**0.008**
Positive	17‍ (27.4%)	13 ‍(11.5%)	

Data expressed as n (%). Significant *p* values are in bold

LNM, lymph node metastasis; mrN Stage, N stage with MRI; mrT stage, T stage with MRI; EMVI, extramural venous invasion; MRF, mesorectal fascia

### Risk factors for LNM

**Fig. 3:**
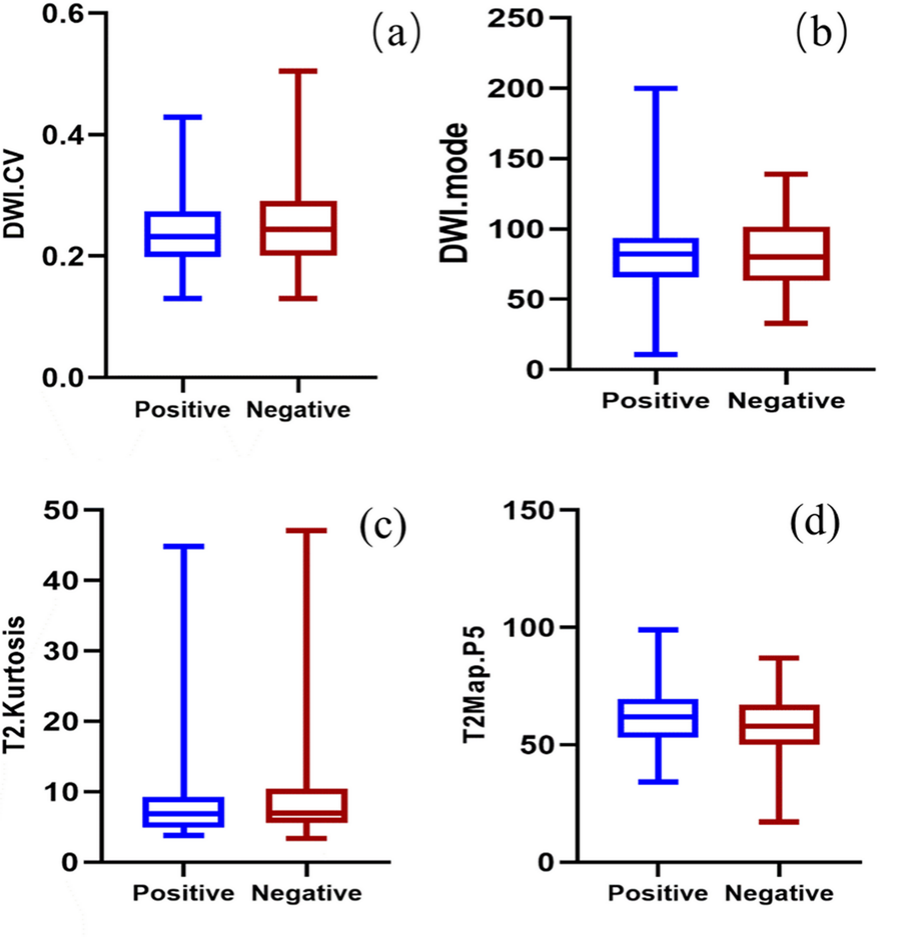
Boxplots of parameters for the non-LNM and LNM groups. **a**_DWI_CV, **b**_DWI_Mode, **c**_T2_Kurtosis, and **d**_T2-map_P5

All the quantitative histogram features were used to draw a ROC curve to obtain cutoff values (see Additional files 1–4) on the basis of LN status. According to the cutoff values, we divided each group into the higher than cutoff value cohort and lower than cutoff value cohort. In these cohorts, _DWI_Skewness, _DWI_Median, _DWI_CV, _DWI_P95, _DWI_Mode, _ADC_Kurtosis, _ADC_CV, _ADC_P5, _ADC_Mode, _T2WI_Kurtosis, _T2WI_CV, and _T2-map_P5 had statistical significance in the evaluation of LNM in rectal cancer. Multivariate logistic regression analysis showed that CA199 level (odds ratio (OR) = 0.104; *p* = 0.009), mrN (OR = 0.124; *p* < 0.001), _T2WI_Kurtosis (OR = 4.101; *p* = 0.010), _T2map_P5 (OR = 0.267; *p* = 0.007), _DWI_CV (OR = 10.135; *p* = 0.038), and _DWI_Mode (OR = 7.744; *p* = 0.038) were independent predictors of LNM, while other covariates were not independent predictors (Table 3, Fig. 3).

**Table 3:** Univariate and multivariate analyses

Parameters	Univariate analysis		Multivariate analysis	
	OR	*p*	OR	*p*
mrN‍ stage	7.686	** < 0.001**	0.124	** < 0.001**
MRF	2.906	**0.009**		
CA199	4.770	**0.012**	0.104	**0.009**
_DWI_Skewness	3.668	**0.026**		
_DWI_Median	2.111	**0.048**		
_DWI_CV	7.253	**0.009**	10.135	**0.038**
_DWI_P95	2.867	**0.011**		
_DWI_Mode	2.326	**0.043**	7.744	**0.038**
_ADC_Kurtosis	0.492	**0.091**		
_ADC_CV	2.742	**0.005**		
_ADC_P5	0.496	**0.029**		
_ADC_Mode	0.402	**0.010**		
_T2WI_Kurtosis	2.495	**0.016**	4.101	**0.010**
_T2WI_CV	2.106	**0.039**		
_T2-map_P5	0.464	**0.017**	0.267	**0.007**

Variables with *p* < 0.05 in the univariate logistic regression analysis were included in the multivariate logistic regression analysis. Significant *p* values are in bold. LNM, lymph node metastasis; Median, 50th percentile in Median histogram; CV, coefficient of variation; P5, 5th percentile; P95, 95th percentile

### Comparison of diagnostic efficiency of different models

**Fig. 4:**
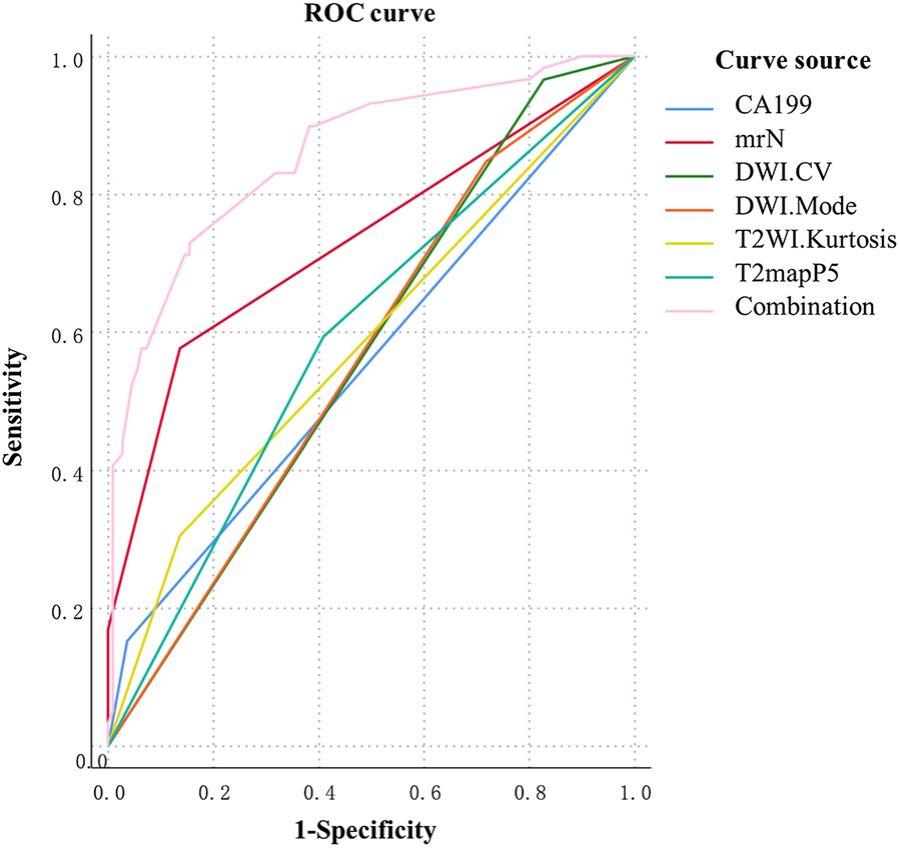
ROC curves for LNM prediction using different parameters and the combination of these parameters

**Fig. 5:**
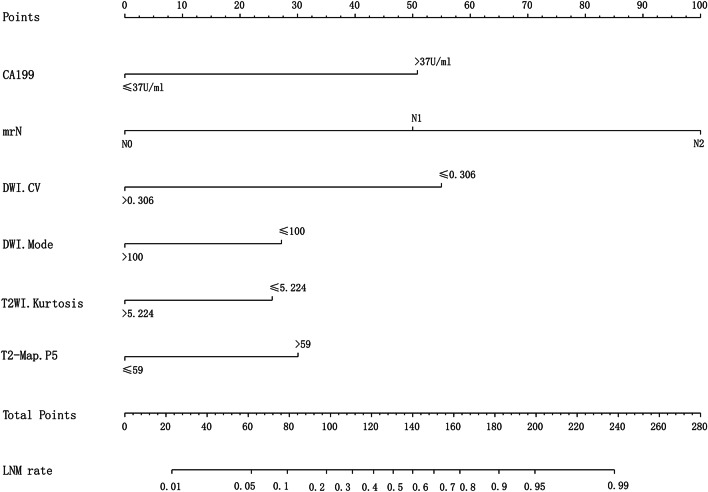
The developed clinical-imaging-histogram nomogram for predicting the probability of LNM. By summing the scores of each point and locating it on the total score scale, the estimated probability of LNM was determined

**Fig. 6:**
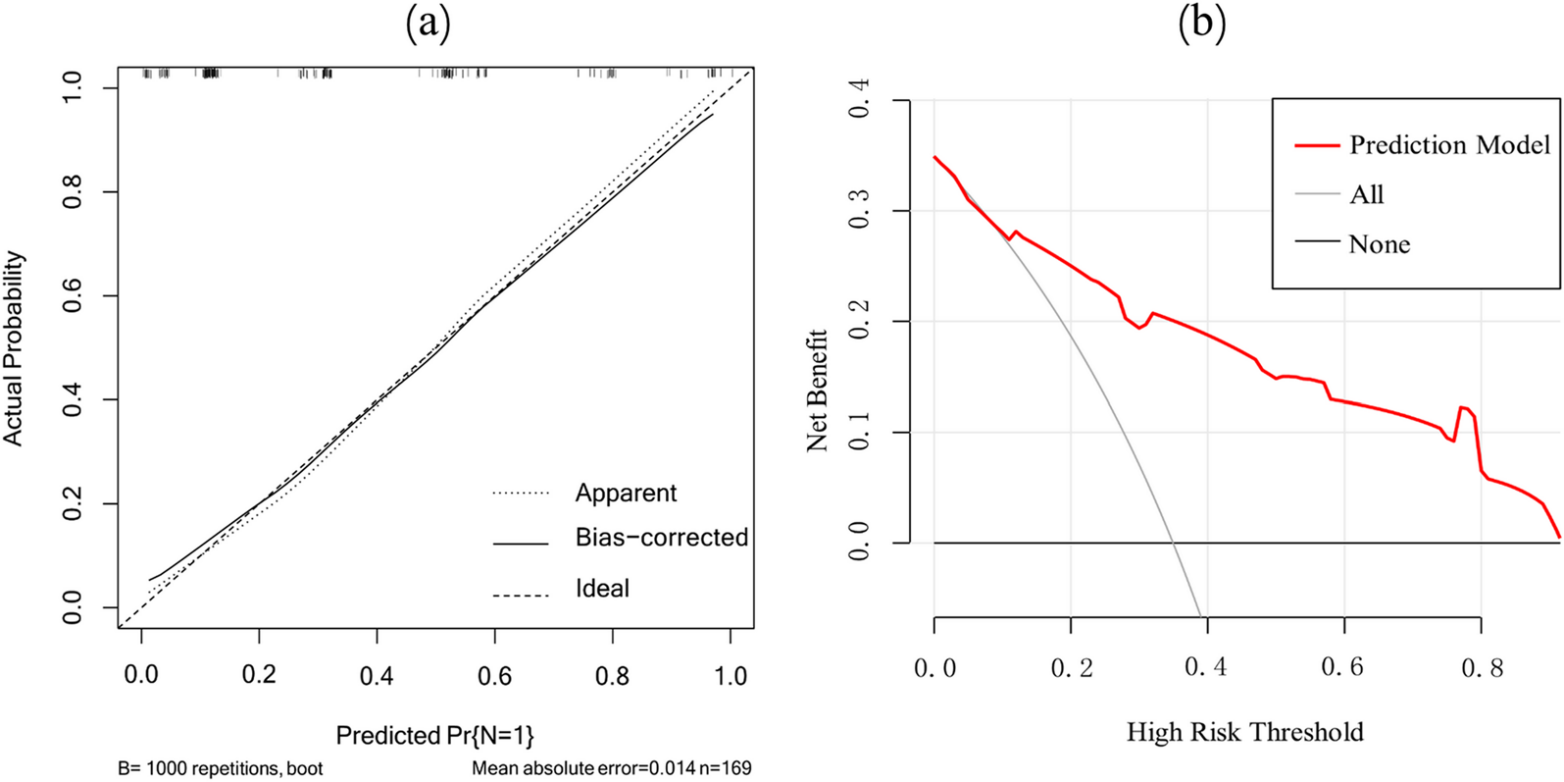
**a** The calibration curves for predicting LNM. The Y axis represents the actual rate of LNM. The X axis represents the predicted probability of LNM. The ideal line represents a perfect prediction by an ideal model. The apparent line represents the performance of the nomogram model, of which a closer fit to the ideal line represents a better prediction. **b** The decision curve analysis for the morphological-histogram nomogram. The red line represents the net benefit of the morphological-histogram model. Across the various threshold probabilities, the morphological-histogram curve showed great net benefit

The diagnostic performance of independent predictors and the combined model were analyzed, respectively, and the results showed that the combined model had the highest AUC value (0.860) (Table 4, Fig. 4). The clinical-imaging-histogram nomogram has good discrimination (Fig. 5). The calibration plots also showed good accordance between the nomogram prediction and the actual outcome for non-LNM and LNM in this data set (Fig. 6a). The decision analysis curve showed satisfactory positive net benefits of the nomogram on most of the threshold probabilities, indicating a favorable potential clinical effect of the model (Fig. 6b).

**Table 4:** Predictive efficacy of the model of four independent predictive factors and the combined model

Parameters	Sensitivity (%)	Specificity (%)	PPV (%)	NPV (%)	AUC
mrN stage	58.1	86.7	70.6	79.0	0.735
CA199	15.3	96.4	69.2	67.9	0.558
_DWI_CV	96.8	19.5	8.3	60.3	0.581
_DWI_Mode	85.5	28.3	22.0	60.4	0.569
_T2WI_Kurtosis	30.6	85	30.9	47.2	0.578
_T2-map_P5	59.7	59.3	44.6	72.8	0.595
Combined model	72.8	85.5	72.9	85.5	0.860

mrN Stage: N stage with MRI; CA199: carbohydrate antigen 199; CV: coefficient of variation; AUC: area under receiver operating characteristic curve; PPV: positive predictive value; NPV: negative predictive value

## Discussion

In this study, we analyzed the histogram features of primary rectal cancer on multi-parametric magnetic resonance images, combined with clinical information and imaging features, and compared the accuracy of doctor diagnosis and the combined model to predict lymph node status; then, based on the combined model, a clinical-imaging-histogram nomogram was constructed. The results showed that imaging features combined with clinical risk factors and medical imaging histogram features have a high value in predicting and diagnosing LMN.

In most cases, the probability of LNM in stage T3 rectal cancer was higher than that in stage T1-2, accounting for 46% [17, 18]. However, there may be differences in prognosis due to different treatment strategies [19]. Therefore, the 2020 NCCN guidelines recommend that the choice of treatment for stage T3 rectal cancer should refer to N stage, and preoperative nCRT should be performed according to the presence or absence of LNM [16]. MRI is the first choice for TNM staging of rectal cancer. Although the diagnostic accuracy of T staging of rectal cancer has reached 88–99% [20], the on-going LNM evaluation accuracy is still less than 80% [21]. 25% of lymph nodes are likely to be over-staged, which will lead to unnecessary preoperative overtreatment, and the possible short-term (such as proctitis) and long-term (such as fecal incontinence, intestinal and genitourinary dysfunction) complications will aggravate the damage caused by the tumor in these patients [22]. Therefore, a definitive diagnosis of lymph node status is a key problem for clinicians in making an appropriate treatment plan for stage T3 patients. In this study, the diagnostic efficiency of radiology features was the same as previous studies, with an accuracy of 71%. The main reason for the deviation in diagnosis may be the diameter of lymph nodes. When the lymph node is smaller than 3 mm, it is difficult to be detected by MRI, which leads to the specificity being 28%, but when it is larger than 9 mm, the sensitivity is 41% [23]. To avoid the above factors, this study evaluated primary tumor lesions to predict the status of lymph nodes and acquired satisfactory results.

In recent years, the application of histograms in the prediction of LNM of primary focus is mainly based on T2WI images. Some studies have shown that histological grade and LNM are positively correlated with kurtosis [24–26]. However, in this study, unlike previous studies, the higher the T2WI kurtosis, the less metastatic, in accordance with the results of Yang et al [8], which demonstrated that the low skewness, kurtosis, energy, and high entropy of T2WI were more meaningful for LNM. We speculated that the results might relate to stage T3 cancer, of which 95.5% were moderately differentiated (Table 1), without differences in differentiation degree. Besides kurtosis, _T2-map_ P5 was associated with LNM in this study. Kurtosis and _T2-map_ P5 were probably related to the fibrous components which contained less water, leading to low T2WI SI and heterogeneity.

Based on previous research on T2WI images, we further incorporated DWI and ADC histogram features into the analysis of primary rectal cancer to predict LNM, which obtained better predictive ability than simple T2WI diagnosis. Each sequence had some features associated with LNM (additional files). In multivariate logistic analysis, there were still four features from three sequences which were independent predictors. They all indicated that primary tumor histogram features were related to LNM. The results showed that the lower the DWI, the higher the ADC SI and the larger the CV, and the less probability of LNM. Thus, our fibrosis hypothesis was further confirmed. At the same histopathological grades, a larger CV and lower tumor cellular density may indicate more fibrosis surrounding the lesion. The existence of fibrosis may contract the blood vessels and lymphatic vessels, restrict cancer cells escaping from the primary focus, and avoid further spread [27].

Finally in association with the doctor's diagnosis, we integrated the laboratory results and the histogram features of the above-mentioned multi-parameter images to verify the influence of the quantitative features of the histogram on the clinical diagnosis. The AUC was 0.860, the sensitivity was 72.8%, and the specificity was 85.5%, which was better than those obtained in previous research [8, 28–33]. We suggest that clinicians should perform TME as soon as possible in patients with stage T3 rectal cancer with normal CA199, negative mrN diagnosis, _DWI_CV >0.306, _DWI_Mode >100 s/mm^2^, _T2WI_Kurtosis >5.224, and _T2-map_P5 ≤59s/mm^2^; to reduce treatment cost and to obtain a more significant survival benefit. On this basis, we propose a clinical-imaging-histogram nomogram, which can be easily used for individual prediction of preoperative LNM in patients with rectal cancer.

Our research has some limitations. First, the study was based on a retrospective analysis carried out in a single center, and there may be biases in patient selection. Secondly, our research only analyzed the histogram features on MRI, and other texture features can also be studied, such as the gray-level co-occurrence matrix. Finally, due to the small sample size, the universal applicability of the nomogram we developed may be limited. Studies with larger samples and multi-agency involvement are required to verify our research results.

## Conclusions

This study shows that the histogram characteristics of primary rectal cancer on multi-parametric MRI sequences are significantly correlated with lymph node status. The histogram quantitative parameters of MRI can increase the accuracy of lymph node evaluation. The clinical-imaging-histogram nomogram can be used as a novel simple scoring system to predict the risk of LNM.

## Supplementary Information

**Additional file 1.** Comparison of T2WI histogram parameters between the LNM and non-LNM groups.

**Additional file 2.** Comparison of DWI histogram parameters between the LNM and non-LNM groups.

**Additional file 3.** Comparison of ADC histogram parameters between the LNM and non-LNM groups.

**Additional file 4.** Comparison of T2-map histogram parameters between the LNM and non-LNM groups.

## Data Availability

The datasets used and/or analyzed during the current study are available from the corresponding author on reasonable request.

## Abbreviations

MRI: Magnetic resonance imaging
LNM: Lymph node metastasis
ROC: Receiver operating characteristic curve
AUC: Area under the curve
nCRT: Neoadjuvant chemoradiotherapy
TME: Total mesorectal excision
ESMO: European Society for Medical Oncology
T2WI: T2 weighted imaging
DWI: Diffusion-weighted imaging
NSA: Number of signal frequency
FOV: The field of view
EMVI: Extramural vascular invasion
MRF: Mesorectal fascia
AJCC: American Joint Commission on Cancer
SI: Signal intensity
NPV: Negative predictive value
PPV: Positive predictive value
CV: Coefficient of variation
MRF: Mesorectal fascia
EMVI: Extramural vascular invasion
CEA: Carcinoma embryonic antigen
