# Correction to: Histogram analysis based on multi-parameter MR imaging as a biomarker to predict lymph node metastasis in T3 stage rectal cancer

**DOI:** 10.1186/s12880-022-00758-w

**Published:** 2022-03-01

**Authors:** Yang Zhou, Rui Yang, Yuan Wang, Meng Zhou, Xueyan Zhou, JiQing Xing, Xinxin Wang, Chunhui Zhang

**Affiliations:** 1 Department of Radiology, Harbin Medical University Cancer Hospital, No. 150, Haping Road, Nangang District, Harbin, 150001 Heilongjiang Province China; 2 Department of Gastrointestinal Medical Oncology, Harbin Medical University Cancer Hospital, Heilongjiang Cancer Institute, No.150 Haping Road, Nangang District, Harbin, 150081 Heilongjiang Province China; 3 School of Technology, Harbin University, Harbin, Heilongjiang Province China; 4 Department of Physical Education, Harbin Engineering University, Harbin, 150001 Heilongjiang Province China

## Correction to: BMC Medical Imaging (2021) 21:176 10.1186/s12880-021-00706-0

Following the publication of the original article [1], the authors requested to amend the article title from “Histogram analysis of diffusion-weighted magnetic resonance imaging as a biomarker to predict LNM in T3 stage rectal carcinoma” to “Histogram analysis based on multi-parameter MR imaging as a biomarker to predict lymph node metastasis in T3 stage rectal cancer”.

The correct title is included in this Correction and has already been updated in the original article.
