# Supplementary material for: Histogram analysis based on multi-parameter MR imaging as a biomarker to predict lymph node metastasis in T3 stage rectal cancer
Source: BMC Med Imaging. 2021 Nov 22;21:176. doi: 10.1186/s12880-021-00706-0 (PMC8609786; doi:10.1186/s12880-021-00706-0)
Supplement: Supplementary file 1 — Additional file 1. Comparison of T2WI histogram parameters between the LNM and non-LNM groups. [file 12880_2021_706_MOESM1_ESM.docx]

**Table. 1** Comparison of T2WI histogram parameters between the LNM and non-LNM groups

| T2WI parameter | Cut-off value | LNM | non-LNM | ***p*** value |
| --- | --- | --- | --- | --- |
| _T2WI_Mean | ≤75.88 | 9（14.5%） | 29（25.7%） | 0.087 |
|  | >75.88 | 53（85.5%） | 84（74.3%） |  |
| _T2WI_Skewness | ≤1.185 | 17（27.4%） | 19（16.8%） | 0.097 |
|  | >1.185 | 45（72.6%） | 94（83.2%） |  |
| _T2WI_Kurtosis | ≤5.224 | 19（30.6%） | 17（15%） | **0.015** |
|  | >5.224 | 43（69.4%） | 96（85%） |  |
| _T2WI_Median | ≤79 | 22（35.5%） | 51（45.1%） | 0.216 |
|  | >79 | 40（64.5%） | 62（54.9%） |  |
| _T2WI_CV | ≤0.346 | 48（77.4%） | 70（61.9%） | **0.037** |
|  | >0.346 | 14（22.6%） | 43（38.1%） |  |
| _T2WI_P5 | ≤55 | 26（41.9%） | 63（55.8%） | 0.080 |
|  | >55 | 36（58.1%） | 50（44.2%） |  |
| _T2WI_P95 | ≤159.1 | 46（74.2%） | 70（61.9%） | 0.101 |
|  | >159.1 | 16（25.8%） | 43（38.1%） |  |
| _T2WI_Mode | ≤76 | 22（35.5%） | 51（45.1%） | 0.216 |
|  | >76 | 40（64.5%） | 62（54.9%） |  |

Data expressed in n (%).Significant p values are in bold. Abbreviations: cut-off value, the best diagnostic cut-off value; LNM, lymph node metastasis; Median, 50th percentile in Median histogram; CV, coefficient of variation; P5, 5th percentile; P95, 95th percentile.
