# Supplementary material for: Histogram analysis based on multi-parameter MR imaging as a biomarker to predict lymph node metastasis in T3 stage rectal cancer
Source: BMC Med Imaging. 2021 Nov 22;21:176. doi: 10.1186/s12880-021-00706-0 (PMC8609786; doi:10.1186/s12880-021-00706-0)
Supplement: Supplementary file 2 — Additional file 2. Comparison of DWI histogram parameters between the LNM and non-LNM groups. [file 12880_2021_706_MOESM2_ESM.docx]

**Table 2.** Comparison of DWI histogram parameters between the LNM and non-LNM groups

| DWI parameter | Cut-off value | LNM | non-LNM | ***p*** value |
| --- | --- | --- | --- | --- |
| _DWI_Mean | ≤85.412 | 44（71%） | 65（57.5%） | 0.079 |
|  | >85.412 | 18（29%） | 48（42.5%） |  |
| _DWI_Skewness | ≤-0.778 | 9（14.5%） | 5（4.4%） | **0.019** |
|  | >-0.778 | 53（85.5%） | 108（95.6%） |  |
| _DWI_Kurtosis | ≤3.069 | 35（56.5%） | 76（67.3%） | 0.156 |
|  | >3.069 | 27（43.5%） | 37（32.7%） |  |
| _DWI_Median | ≤91s/ | 36（58.1%） | 89（78.8%） | **0.004** |
|  | >91s/ | 26（41.9%） | 24（21.2%） |  |
| _DWI_CV | ≤0.306 | 60（96.8%） | 91（80.5%） | **0.003** |
|  | >0.306 | 2（3.2%） | 22（19.5%） |  |
| _DWI_P5 | ≤35.2 | 8（12.9%） | 24（21.2%） | 0.172 |
|  | >35.2 | 54（87.1%） | 89（78.8%） |  |
| _DWI_P95 | ≤128.1 | 53（85.5%） | 76（67.3%） | **0.009** |
|  | >128.1 | 9（14.5%） | 37（32.4%） |  |
| _DWI_Mode | ≤100 | 53（85.5%） | 81（71.7%） | **0.039** |
|  | >100 | 9（14.5%） | 32（28.3%） |  |

Data expressed in n (%).Significant p values are in bold. Abbreviations: cut-off value, the best diagnostic cut-off value; LNM, lymph node metastasis; Median, 50th percentile in Median histogram; CV, coefficient of variation; P5, 5th percentile; P95, 95th percentile .
