# Supplementary material for: Histogram analysis based on multi-parameter MR imaging as a biomarker to predict lymph node metastasis in T3 stage rectal cancer
Source: BMC Med Imaging. 2021 Nov 22;21:176. doi: 10.1186/s12880-021-00706-0 (PMC8609786; doi:10.1186/s12880-021-00706-0)
Supplement: Supplementary file 3 — Additional file 3. Comparison of ADC histogram parameters between the LNM and non-LNM groups. [file 12880_2021_706_MOESM3_ESM.docx]

**Table 3.** Comparison of ADC histogram parameters between the LNM and non-LNM groups

| ADC parameter | Cut-off value | LNM | non-LNM | ***p*** value |
| --- | --- | --- | --- | --- |
| _ADC_Mean | ≤0.9×10^-3^mm^2^/s | ‍3（4.8%） | 13（11.5%） | 0.143 |
|  | >0.9×10^-3^mm^2^/s | 59（95.2%） | 100（88.5%） |  |
| _ADC_Skewness | ≤0.561 | 13（30%） | 35（40%） | 0.156 |
|  | >0.561 | 49（70%） | 78（60%） |  |
| _ADC_Kurtosis | ≤3.137 | 8（12.9%） | 30（26.5%） | **0.036** |
|  | >3.137 | 54（87.1%） | 83（73.5%） |  |
| _ADC_Median | ≤0.9×10^-3^mm^2^/s | 9（14.5%） | 29（25.7%） | 0.087 |
|  | >0.9×10^-3^mm^2^/s | 53（85.5%） | 84（74.3%） |  |
| _ADC_CV | ≤0.218mm^2^/s | 23（37.1%） | 20（17.7%） | **0.004** |
|  | >0.218mm^2^/s | 39（62.9%） | 93（82.3%） |  |
| _ADC_P5 | ≤0.7×10^-3^mm^2^/s | 26（41.9%） | 67（59.3%） | **0.028** |
|  | >0.7×10^-3^mm^2^/s | 36（58.1%） | 46（40.7%） |  |
| _ADC_P95 | ≤1.6×10^-3^mm^2^/s | 41（66.1%） | 67（59.3%） | 0.373 |
|  | >1.6×10^-3^mm^2^/s | 21（33.9%） | 46（40.7%） |  |
| _ADC_Mode | ≤0.8×10^-3^mm^2^/s | 16（25.8%） | 49（43.4%） | **0.022** |
|  | >0.8×10^-3^mm^2^/s | 46（74.2%） | 64（56.6%） |  |

Data expressed in n (%).Significant p values are in bold. Abbreviations: cut-off value, the best diagnostic cut-off value; LNM, lymph node metastasis; Median, 50th percentile in Median histogram; CV, coefficient of variation; P5, 5th percentile; P95, 95th percentile.
