# Supplementary material for: Histogram analysis based on multi-parameter MR imaging as a biomarker to predict lymph node metastasis in T3 stage rectal cancer
Source: BMC Med Imaging. 2021 Nov 22;21:176. doi: 10.1186/s12880-021-00706-0 (PMC8609786; doi:10.1186/s12880-021-00706-0)
Supplement: Supplementary file 4 — Additional file 4. Comparison of T2-map histogram parameters between the LNM and non-LNM groups. [file 12880_2021_706_MOESM4_ESM.docx]

**Table 4.** Comparison of T2-map histogram parameters between the LNM and non-LNM groups

| T2-map parameter | Cut-off value | LNM | non-LNM | ***p*** value |
| --- | --- | --- | --- | --- |
| _T2-map_Mean | ≤92.632 | 25（40.3%） | 61（54%） | 0.084 |
|  | >92.632 | 37（59.7%） | 52（46%） |  |
| _T2-map_Skewness | ≤1.304 | 22（35.5%） | 35（31%） | 0.543 |
|  | >1.304 | 40（64.5%） | 78（69%） |  |
| _T2-map_Kurtosis | ≤7.919 | 44（71%） | 73（64.6%） | 0.394 |
|  | >7.919 | 18（29%） | 40（35.4%） |  |
| _T2-map_Median | ≤81.5 | 20（32.2%） | 51（45.1%） | 0.097 |
|  | >81.5 | 42（67.8%） | 62（54.9%） |  |
| _T2-map_CV | ≤0.45 | 56（90.3%） | 89（78.8%） | 0.052 |
|  | >0.45 | 6（9.7%） | 24（21.2%） |  |
| _T2-map_P5 | ≤59 | 25（40.3%） | 67（59.3%） | **0.016** |
|  | >59 | 37（59.7%） | 46（40.7%） |  |
| _T2-map_P95 | ≤151 | 30（48.4%） | 46（40.7%） | 0.327 |
|  | >151 | 32（51.6%） | 67（59.3%） |  |
| _T2-map_Mode | ≤76 | 25（40.3%） | 62（54.9%） | 0.066 |
|  | >76 | 37（59.7%） | 51（45.1%） |  |

Data expressed in n (%).Significant p values are in bold. Abbreviations: cut-off value, the best diagnostic cut-off value; LNM, lymph node metastasis; Median, 50th percentile in Median histogram; CV, coefficient of variation; P5, 5th percentile; P95, 95th percentile.
